# Multi-Scale Motility Amplitude Associated with Suicidal Thoughts in Major Depression

**DOI:** 10.1371/journal.pone.0038761

**Published:** 2012-06-12

**Authors:** Premananda Indic, Greg Murray, Carlo Maggini, Mario Amore, Tiziana Meschi, Loris Borghi, Ross J. Baldessarini, Paola Salvatore

**Affiliations:** 1 Department of Neurology, University of Massachusetts Medical School, Worcester, Massachusetts, United States of America; 2 Faculty of Life and Social Sciences, Swinburne University of Technology, Hawthorn, Victoria, Australia; 3 Section of Psychiatry, Department of Neuroscience, University of Parma, Parma, Italy; 4 Section of Internal Medicine, Department of Clinical Sciences, University of Parma, Parma, Italy; 5 International Consortium for Psychotic and Bipolar Disorders Research, McLean Hospital, Belmont, Massachusetts, United States of America; 6 Department of Psychiatry, Harvard Medical School and McLean Hospital, Boston, Massachusetts, United States of America; University of South Florida, United States of America

## Abstract

Major depression occurs at high prevalence in the general population, often starts in juvenile years, recurs over a lifetime, and is strongly associated with disability and suicide. Searches for biological markers in depression may have been hindered by assuming that depression is a unitary and relatively homogeneous disorder, mainly of mood, rather than addressing particular, clinically crucial features or diagnostic subtypes. Many studies have implicated quantitative alterations of motility rhythms in depressed human subjects. Since a candidate feature of great public-health significance is the unusually high risk of suicidal behavior in depressive disorders, we studied correlations between a measure (*vulnerability index* [*VI*]) derived from multi-scale characteristics of daily-motility rhythms in depressed subjects (*n = 36*) monitored with noninvasive, wrist-worn, electronic actigraphs and their self-assessed level of suicidal thinking operationalized as a wish to die. Patient-subjects had a stable clinical diagnosis of bipolar-I, bipolar-II, or unipolar major depression (*n = *12 of each type). *VI* was associated inversely with suicidal thinking (*r* =  –0.61 with all subjects and *r* =  –0.73 with bipolar disorder subjects; both *p*<0.0001) and distinguished patients with bipolar versus unipolar major depression with a *sensitivity* of 91.7% and a *specificity* of 79.2%. *VI* may be a useful biomarker of characteristic features of major depression, contribute to differentiating bipolar and unipolar depression, and help to detect risk of suicide. An objective biomarker of suicide-risk could be advantageous when patients are unwilling or unable to share suicidal thinking with clinicians.

## Introduction

The scientific and diagnostic validity of proposed clinical disorders can be supported by biological markers [1]. Such indicators can lead to pathophysiological or etiological theories, may foster early diagnosis, and support improved prognosis and treatment [2]. Development of such markers is particularly urgent for major mood disorders, which are prevalent, often start early in life with multiple recurrences, and affect nearly 10% of the general population in a lifetime [3]. They are leading international causes of disability and carry risk of premature mortality from suicide and other violence, as well as adverse outcomes of comorbid medical illnesses [3], [4]. These risks are particularly high in bipolar (manic-depressive) mood disorders [5].

The search for potential biological markers of clinical depression probably has been hindered by currently employed, standardized diagnostic criteria. In particular, it is recognized that diagnoses of major depressive disorder based on the influential DSM-IV taxonomy are broad and limited in both specificity and longitudinal stability [6], [7]. Furthermore, excessive focus on changes in mood as a hallmark, as well as the assumption that clinical depression is largely homogeneous may be misleading and probably have contributed to the lack of credible biomarkers [8]. A potentially more effective strategy is to focus on particular aspects of clinical depression and bipolar disorders. These include diagnostic subtypes as well as particularly striking, characteristic, and clinically important features. Candidate targets include more reliable and earlier differentiation of recurrent unipolar major depression from bipolar disorder and its subtypes, I (with mania) and II (depression with hypomania), as well as improved prediction of suicide risk [9], [10].

The search for biomarkers of suicide is particularly challenging as vulnerability to suicidal behaviors is a highly complex phenomenon. Suicide risk is most likely mediated by a genetic diathesis interacting with environmental and psychosocial risk factors as well as epigenetic mechanisms that alter the function of neuronal circuits early in life, and exert effects persisting throughout adulthood [11], [12], [13].

Relevant biological, psychosocial and environmental risk factors for suicidal behaviors include maternal stress during pregnancy, restricted fetal growth, birth complications and deprivation of normal parental care during infancy as well as precocious adversities such as sexual and physical abuse, neglect, parental loss and family discord. Decades of research have documented abnormalities in the hypothalamic-pituitary-adrenal axis (HPA), as well as the noradrenergic and serotonergic systems with altered stress-sensitivity responses reported in individuals with suicidal behavior as well as during major depression [11], [14], [15]. In addition, early exposure to violence, substance abuse, and head injury have proven to mold stress responses in the brain and thereby affect the risk of suicide in major depression [11], [13].

Recent studies have sought evidence of a neurobiological contribution to suicide risk based on an endophenotype strategy aimed at identifying quantitative measures that reflect stable, genetically-influenced changes in brain function [16], [17]. Candidate endophenotypes include impulsive-aggressive personality traits, deficits in risk-assessment, and possibly related disturbances in prefrontal cortical functioning and in neuroendocrine responses [11], [13], [17].

A promising alternative to a traditional focus on changes in mood in major depression to better identify core aspects for defining potential biomarkers, is psychomotor activity. This objective and quantifiable factor has been of interest for depressive disorders for more than a century. Notably, in the 1890s, Wilhelm Weygandt described the clinical value of structured self-training for voluntary activation of certain groups of muscles to induce specific changes of mood, based on methods employed by theater actors [18]. More recently, many studies have implicated quantitative alterations in circadian and ultradian rhythms of psychomotor activity associated with clinical affective states [19], [20]. Motor activity can be conveniently and noninvasively recorded for prolonged periods in human subjects with wrist-worn, microprocessor-controlled, piezoelectric actigraphic devices that provide objective, quantitative evaluation of motility levels as well as their dynamic changes [21].

Actigraphy has documented reduced total activity or blunted amplitude of daily motility rhythms in major depression [22], often with circadian phase-advances. Such alterations have been particularly striking among subjects diagnosed with bipolar disorder during mania, depression or shortly before or after acute episodes of illness, as well as in mild or subsyndromal morbid phases [19], [20], [23]. Also reported are phase-delays of circadian motility cycles in major depression, bipolar depression, and seasonal affective disorders [24], [25]. Some changes persist after clinical recovery from depression or mania, and so may serve as biomarkers of stable traits, and not only as covariates of current mood-states [22], [26].

Based on wavelet analysis of motility data from patients with mood disorders, we recently reported a scaling behavior of amplitudes of the rhythms at multiple time-scales ranging from minutes to hours. The amplitude of these rhythms exhibited a long-tail distribution at time-scales up to 2 hours [27]. Such a long-tail amplitude distribution is characteristic of nonlinear complex systems [28]. Based on application of a Gamma function to the distribution of amplitudes (see Methods), we derived a novel *vulnerability index* (*VI*), by integrating the shape parameters obtained by fitting amplitude distribution to a Gamma function up to scales of 2 hours. The *VI* could distinguish between: (i) currently healthy subjects at high- versus low-risk for type-I bipolar disorder, (ii) clinically stable or euthymic type-I bipolar disorder patients from healthy controls, (iii) and among clinical states in type-I bipolar disorder patients, based on higher values of the index with higher ratings of mania and lower values related to higher ratings of depression [27].

In the present study we investigated correlations between *VI*, as an objective measure of multi-scale characteristics of motility rhythms, and self-assessed psychopathology including suicidal thinking operationalized as a wish to die, in human subjects monitored with actigraphic devices during an episode of major depression. Our primary aim was to explore whether suicidal thinking might correlate with this measure of psychomotor activity. A secondary aim was to test the ability of *VI* to distinguish between patients diagnosed clinically with bipolar versus unipolar major depression.

## Methods

### Ethics Statement

All subjects provided written informed consent for aggregate and anonymous reporting of data arising from their clinical and actigraphic assessments. Study was approved by the Ethics Committee of the University of Parma Medical Center, in full accordance with international standards for the ethical use of human subjects in research.

### Clinical Procedures

Participants were recruited as outpatients at the Section of Psychiatry of the University of Parma, Italy. Their DSM-IV psychiatric diagnoses [7] were supported by semi-structured interviews conducted repeatedly by research psychiatrists at intake and at follow-up assessments over 7.43±2.24 years.

Clinician ratings of subject symptom-severity were made at study entry with the Young Mania Rating Scale (YMRS) and the Hamilton Depression Rating Scale (HDRS) as reported previously [26]. In addition, a total of 29 statements, (details are available on request), describing subjective experiences of affective psychopathology in layman’s language, were rated by study-participants as 0–10 on a 10 cm visual-analog scale anchored by “Not at all" and “Very much" [29], once-daily, at bedtime, on the three consecutive days of actigraphic monitoring, and scores (1 cm-increments) averaged across the three daily assessments were statistically analyzed.

The 29 self-rated statements were devised and tested in a decade-long study by a clinical-research consensus-workgroup at the University of Parma seeking to capture potentially relevant realms of subjective affective psychopathology, based on items of the Bonn Scale for the Assessment of Basic Symptoms [30] and the Hamburg Scale for mixed manic-depressive states and bipolar illness [18], [31]. Relevant areas identified by this process and employed in the present study included vital drive or wish to live, suicidal thinking or wish to die, depression, irritable mood (dysphoria), anxiety, as well as unusual body-perception experiences (cœnesthesias) and neurovegetative (autonomic nervous system) symptoms.

In particular, the statement of primary interest here was statement 15,"Ho sentito il desiderio di morire o di essere morto" (“I felt the wish to die or be dead" **–** *translated by PS*). The remaining 28 statements measure 6 constructs: depression and vital drive described in relation to psychomotor activity in statements 2 & 3, to mood (statements 6 & 7), to train of thoughts (statements 9 & 10), to verbal flow (statements 11 & 13), to hedonic drive (statements 16 & 17), to libido (statements 18 & 19), and to thought content (statements 28 & 29); dysphoria described in relation to psychomotor activity in statements 1 and 5, to mood (statement 4), to thought content (statement 8), and to verbal behavior (statement 12); anxiety described in statement 14; unusual body-perception experiences (coenesthesias) described in statements 20–23, and neurovegetative (autonomic nervous system) symptoms in statements 24–27.

The patient-subjects had a stable DSM-IV [7] diagnosis of bipolar-I, bipolar-II, or unipolar major depressive disorder (*n* = 12 subjects of each type). All subjects were in current episodes of DSM-IV major depression with minimum symptom ratings of 26 (moderately severe) on the standard Hamilton Depression Rating Scale (17-item HDRS), and ≤4 (minimal score) on the Young-Mania Rating Scale (YMRS) [26]. No subject had a past or recent history of suicidal attempts or deliberate self-harmful acts.

### Actigraphy Data Analysis

Motility of each subject was recorded continuously for 72 hours using an actigraphic device (AMI-128K Mini-Motionlogger® Actigraph, Ambulatory Monitoring, Inc. [AMI], Ardsley, NY, USA), worn on the non-dominant wrist continuously, 24 hours a day under naturalistic, ambulatory conditions. The device detected and recorded piezoelectric signals arising from body movements, sampling at 32 Hz and integrating digitized data in 6-minute epochs. The motility data contain rhythms at multiple time-scales. As described previously [27], the amplitude of such rhythms at various time-scales was obtained using a continuous wavelet transform by convolution of the acquired data with a scaled and translated version of a Morlet wavelet as a mother wavelet. Wavelet transform is a powerful tool for estimating the amplitudes from multi-scale data. Amplitude of the rhythms at short time-scales appeared to be random; however its distribution exhibited a long-tail characteristic with a scaling behavior at time-scales up to 2 hours. Long-tail distribution is considered as a signature of nonlinear complex systems near critical points, and the Gamma function is used to characterize such distribution [28]. Hence we characterized the distribution of amplitudes at each scale using a Gamma function fit. The shape parameter obtained by the fit was integrated up to ∼2 hours to derive *VI*.

### Statistical Analysis

We compared subjects with DSM-IV-based diagnoses of type-I or -II bipolar disorder or recurrent unipolar major depressive disorder in a current episode of major depression. We based statistical comparisons on ANOVA (*F*) methods for continuous variables, and contingency tables (χ^2^ or Fisher exact-*p*) for categorical factors, with defined degrees-of-freedom (*df*). We used multivariate logistic regression modeling to predict diagnostic group from clinical and actigraphic factors. Based on multiple linear regression modeling, we analyzed the relationship between the objective measure *VI* and the subjective phenomenological measures as well as clinical and diagnostic factors. Bayesian characteristics were computed to distinguish bipolar depression from unipolar depression. Averages are means with standard deviations (±SD) unless stated otherwise. Analyses are based on commercial statistical programs (Stata-9®, Stata Corp., College Station, TX; Statview-5®, SAS Institute, Cary, NC).

## Results

### Characteristics of Patients with Major Depression

**Figure 1:**
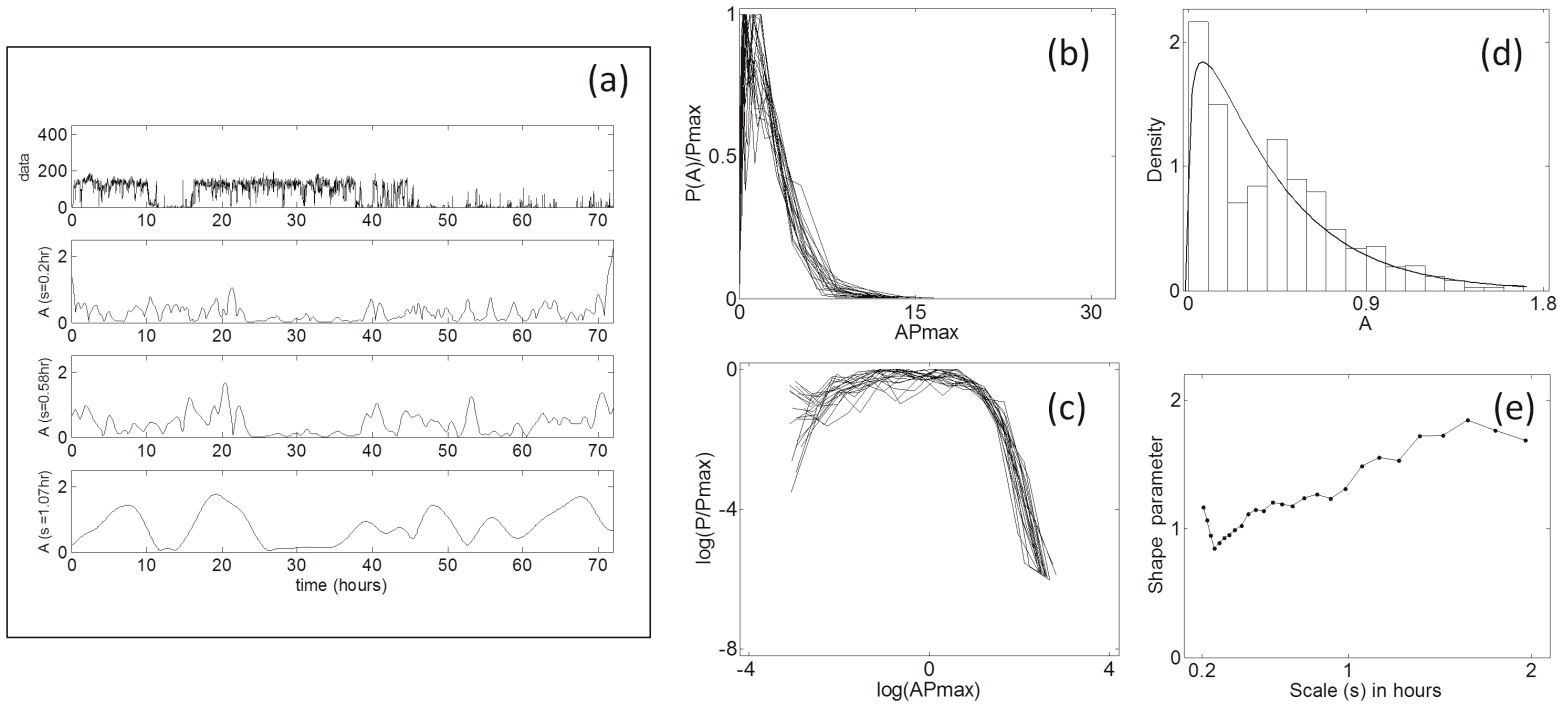
Characterization of multi-scale amplitude of motility data by wavelet analysis from a representative subject with major depression. (**a**) Raw data in arbitrary units (a.u), with amplitudes of rhythms detected at three time-scales. Subject shows a disrupted activity profile due to disturbed sleep/wake cycle which is common in depressed subjects. (**b**) Rescaled probability distribution of amplitude obtained by rescaling using *P_max_* to provide unit area of amplitude at a range of time-scales up to 2 hours. (**c**) The same data, log-transformed and showing a long-tail, which share most of the values of log (*AP_max_)*. (**d**) Gamma-distribution function fit of the amplitude distribution at a scale of 0.58 hours. The goodness-of-fit is determined by Akaike information criteria in comparison with other distributions (20). (**e**) The shape parameter obtained from the Gamma-function fit decreases at short time-scales and increases at long time-scales. *VI* is obtained by finding the area under the curve.

Representative motility data at selected time-scales obtained from wavelet analysis along with the distribution of observed amplitudes are shown in Figure 1. Although these subjects had a disrupted activity profile due to irregular sleep/wake behavior, the distribution of their motility amplitudes at multiple time-scales exhibited a long-tail distribution.

Among the 36 subjects, diagnostic groups did not differ in current age (*F* [*df* = 2; 33] = 1.24, *p* = 0.30), sex-distribution (χ^2^ [df = 2] = 3.56, *p* = 0.23), or baseline HDRS-scored depression-severity (*F* [df = 2; 33] = 1.00, *p* = 0.38; Table 1). However, mean *VI* scores ranked: bipolar-II < bipolar-I < unipolar major depressive disorders (Table 1). Multinomial logistic regression modeling predicted diagnostic group from *VI* scores highly significantly when unipolar major depressive disorder was the reference group (χ^2^ [*df* = 2] = 24.3, *p*<0.001, Cox and Snell pseudo-*r* ^2^ = 0.49). Unipolar depressed patients differed from both the bipolar-I (Wald statistic = 4.35, *p* = 0.04) and bipolar-II cases (Wald statistic = 10.9, *p*<0.001). When regression modeling considered bipolar-I disorder patients as the reference group, *VI* distinguished between bipolar disorder types I and II (Wald statistic = 5.82, *p* = 0.02).

**Table 1:** Characteristics of patients with major depression by DSM-IV diagnostic types.

Characteristics	All Cases	Bipolar-I	Bipolar-II	Unipolar	κ^2^ or *F*	*p*-value
Cases (n)	36	12	12	12	–	–
Women (%)	75.0	75.0	58.3	91.7	3.55	0.23
Age (years)	46.1±12.7	41.7±10.3	49.6±14.6	47.0±12.4	1.24	0.30
HDRS depression rating	30.4±2.21	30.5±2.28	30.9±1.88	29.7±2.42	1.00	0.379
Vulnerability index (*VI*) score	2.84±0.94	2.85±0.76	2.04±0.60	3.63±0.70	16.0	<0.0001
*Self-ratings*						
Vital drive (wish to live)	12.0±12.4	4.94±8.92	12.2±12.4	18.7±12.3	4.46	0.02
Suicidal thoughts (wish to die)	2.20±2.44	1.76±1.95	3.53±2.42	1.28±2.50	3.18	0.05
Depression	25.5±15.6	24.0±17.7	31.6±16.3	20.8±11.6	1.54	0.23
Unusual bodily sensations	4.76±7.74	5.70±11.3	3.80±4.40	4.84±6.50	0.16	0.85
Anxiety	2.55±3.38	1.72±2.56	3.03±3.36	2.90±4.17	0.54	0.59
Dysphoria	6.80±5.90	6.01±8.20	7.98±4.51	6.31±4.52	0.38	0.69
Autonomic symptoms	6.44±6.52	3.38±4.50	8.86±6.86	7.10±7.13	2.39	0.85
*Total morbidity* score	60.3±39.8	47.6±38.7	72.3±40.8	61.9±39.6	1.08	0.35
Clinician-rated dysphoria (%)	63.9	41.7	100.0	50.0	10.4	0.005

Statistical tests for diagnostic differences: for continuous variables, one-way ANOVA [*df* = 2; 33] (*F*); for categorical values, contingency tables [*df* = 2] (χ^2^). Bonferroni-adjusted criterion *p*<0.004 (0.05/13). Data are means ±SD unless stated otherwise. HRSD: Hamilton Rating Scale for Depression.

Self-rated as well as clinician-rated severity of current depression was similar across diagnoses, whereas ratings of suicidal thoughts followed an opposite ranking to that for *VI*, with suicidal ideation scores ranking: bipolar-II > bipolar-I > unipolar major depressive disorders (Table 1). Clinician-based assessments found the prevalence of dysphoria (irritable mood) during depression to rank: bipolar-II > unipolar > bipolar-I diagnosis; bipolar-I disorder subjects also scored significantly lower in self-rated vital drive or wish to live than unipolar major depressive disorder subjects (Table 1).

### Relationship of *VI* and Self-assessed Phenomenological Measures

**Figure 2:**
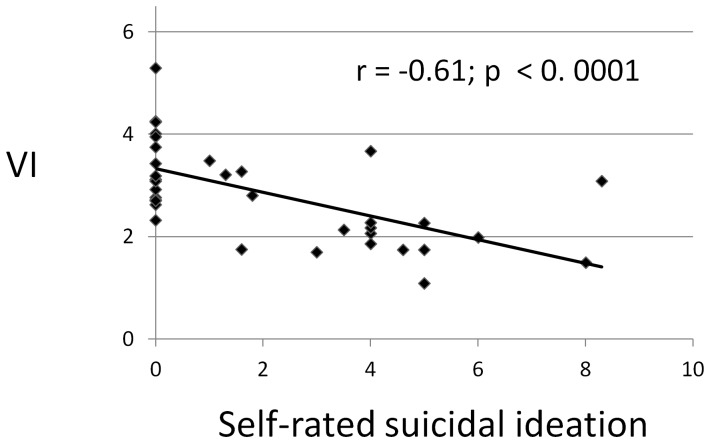
Relationship of *VI* to self-rated suicidal ideation in subjects with major depression. The negative correlation between *VI* and self-rated suicidal ideation (*r* = –0.61) is highly significant (*p*<0.0001) among depressed subjects (*n* = 36 bipolar I and II and unipolar major depressive disorder subjects).

Based on linear regression modeling of the relationship between the objective measure *VI* and seven subjective phenomenological measures, we found that: [i] the only factor strongly related to *VI* (lower scores) was self-rated suicidal thinking, across diagnoses (slope [β] factor =  –0.233, *t* = –0.45, *p*<0.0001, based on Pearson’s correlation, *r* = –0.61 [Figure 2]); [ii] no other factor was associated with *VI* scores, including subjective self-assessments of current depression, vital drive or wish to live, dysphoria, anxiety, unusual body-perception experiences (cœnesthesias), neurovegetative (autonomic nervous system) symptoms, and total psychopathology (for the last of which β = –0.0006, *t* = –0.14, *p* = 0.89, *r* = –0.025); [iii] the association of lower *VI* with higher suicidal self-ratings remained significant when modeling included other factors (sex, diagnosis, clinician-rated dysphoria, self-rated depression, and self-ratings of vital drive, dysphoria, anxiety, unusual body-perception experiences [cœnesthesias], and autonomic-neurovegetative symptoms); [iv] a multiple linear regression model indicated that a strong association of *VI* score with lower self-ratings of suicidal ideation but not with self-reported depressed mood, remained even with other key factors added, and that *VI* score was more associated with unipolar than bipolar (I or II) diagnosis (Table 2). Vital drive itself also was independently associated with *VI* scores [β = +0.022, *t* = 2.27, *p* = 0.03, *r* = +0.292]).

**Table 2:** Multiple linear regression model of factors associated with higher *vulnerability index* (*VI*) scores.

Factors	Slope (β) [95%CI]	*t*-score	*p*-value
Unipolar > bipolar depression	0.946 [+0.469 to+1.42]	4.04	<0.0001
*Less* self-reported suicidal ideation	–0.199 [–0.302 to –0.096]	3.94	<0.0001
Self-rated depression	+0.005 [+0.011 to+0.021]	0.66	0.512

*VI* score was associated more strongly with unipolar than bipolar (I or II) depression and with lower self-ratings of suicidal ideation or wish to die, but was not associated with self-reported depressed mood. Factors not associated with *VI* score included sex, age, clinician-rated dysphoria, and self-ratings of dysphoria, vital drive, anxiety, unusual bodily experiences, autonomic symptoms, or total morbidity.

A post-hoc statistical power analysis of multiple linear regression accounting for number of predictors *N* = 10, observed R*^2^* = 0.37, probability level *α* = 0.05 and sample size *n* = 36 found an observed statistical power of *π* = 0.77 that is close to the standard for adequacy of power of *π* = 0.80 conventionally established to avoid probability of type II error or false negative rate, *β* = 0.20.

**Figure 3:**
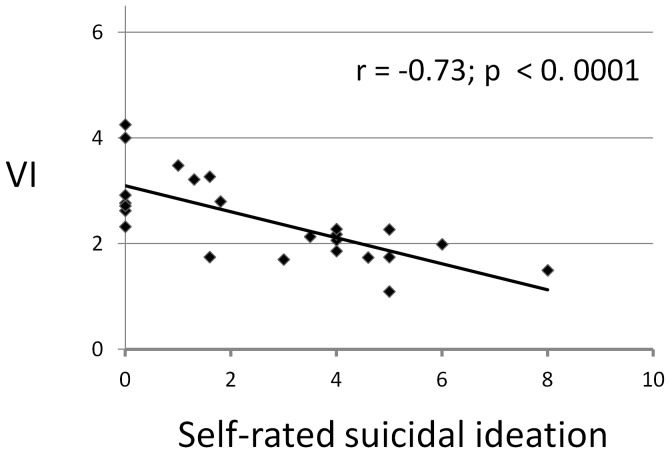
Relationship of *VI* to self-rated suicidal ideation in bipolar-illness subjects with major depression. The negative correlation between *VI* and self-rated suicidal ideation (*r* = –0.73) is highly significant (*p*<0.0001) among bipolar depressed subjects (*n* = 24 bipolar I and II disorder subjects).

We also computed Bayesian characteristics for detecting bipolar depression and distinguishing it from unipolar depression, based on a criterion value for elevated *VI* at ≥3.0. This test-criterion yielded substantial and encouraging probabilities for *sensitivity* (probability of detecting unipolar depression [“true-positive" test result]) of 91.7%, *specificity* (probability of excluding bipolar depression) of 79.2%, and *positive predictive value* (probability of identifying unipolar depression among all cases with elevated *VI*) of 68.8%. We also considered *VI* scores and self-rated suicidal thinking in bipolar disorder patients since most (n = 10/12) unipolar depressed subjects reported a lack of suicidal thoughts, in accord with the lower suicidal risk among moderately depressed outpatients with unipolar disorder [32]. We found a strong correlation between *VI* scores and self-rated suicidal thinking in the bipolar depressed patients (Figure 3).

## Discussion

The strong negative association between *VI* and self-rated suicidal thinking or wish to die, and its positive association with self-rated vital drive or wish to live suggest that *VI* may be associated specifically with contrasting forces that often confront one other in mood disorder patients–the wish to die and the wish to live [33]. The lack of correlations between *VI* and other subjective phenomenological measures, including depressed mood, dysphoria, anxiety, unusual body-perception experiences (cœnesthesias), and autonomic-neurovegetative symptoms suggests at least two levels of subjective psychopathology in depression: [a] experiences of decreased fundamental vitality rooted in psychomotor activity (reflected in *VI* scores), and [b] more cognitive experiences of hopelessness-helplessness (self-rated depression) or of anguish, fear, anger and distress (self-rated anxiety and dysphoria) associated with abnormal somatic experiences (self-rated autonomic-cœnesthetic symptoms). A similar perspective on descriptive mapping of human emotional life into levels of particular feeling-states and emotions was articulated nearly a century ago by Max Scheler [34]. Such an approach, with a focus on specific elements of emotional life, might provide a useful theoretical framework for future research, including psychobiological studies, in mood disorders [35].

In spite of comparable levels of clinician-rated depression across diagnoses, there were relevant differences in depressive phenomenology in bipolar disorders versus unipolar major depressive disorder subjects. In particular, the higher scores for self-rated suicidal ideation found in bipolar-II disorder subjects accord with recent reports of high risks of suicide and attempts in both bipolar I and II disorder patients, especially in relation with intense impulsivity and dysphoria [32]. The greater prevalence of clinician-rated dysphoria in the bipolar-II subjects versus those with either bipolar-I or unipolar major depressive disorder also accords with the same view of type-II bipolar disorder as an illness underlined by impulsive-emotional dysregulation and possible relations with other clinical syndromes characterized by impulsive-emotional dyscontrol [36].

In addition, bipolar-I disorder subjects scored significantly lower in self-rated vital drive or wish to live than unipolar major depressive disorder subjects. This observation of a diminished self-perceived level of vigor and vital drive, along with decreased motility, confirms the greater likelihood in bipolar depression of psychomotor retardation, lethargy, and lack of vitality compared to unipolar depression [19], [37], [38]. Such phenomenological differences between unipolar and bipolar depressive episodes may be related to neurobiological mechanisms including those underlying attentional control, visuospatial and sensory processing, and emotional regulation [39], [40], [41], differences in central monoaminergic systems [42], [43], and altered electroencephalographic patterns during sleep phases [44].

In light of the findings reported, we also hypothesize that theoretical models for suicide research and prevention might profit by shifting focus from monitoring of experiences of disturbed affect and emotions to consideration of fundamental aspects of vitality and psychomotor activity. Relevant considerations might include two crucial clinical phenomena, potentially emerging during major depression and long recognized in classical psychopathology as associated with increased vulnerability to suicide. The first phenomenon is a time during the process of recovery from depression when psychomotor inhibition resolves before depressed mood. This view predicts that suicidal impulses are more likely to be acted upon at the particularly dangerous time of partial recovery [45]. The second phenomenon involves the presence of mixed affective states during bipolar depressive phases that can create a highly explosive mixture of deep sadness, rage and despair as well as psychomotor arousal associated with impulsive aggression leading to self-harm or death [18].

Recent research on the neurobiology of suicidal behaviors indicates that impulsive aggression as well as impaired judgment or deficient risk assessment may constitute core endophenotypes of suicidality [11], [13], [17]. In addition, an aggressive diathesis can be viewed as an imbalance between the diminished effects of descending control systems in the orbital frontal and anterior cingulate cortices, and excessive emotional and aggressive drives arising from limbic elements including the amygdala and insula [46]. The modulation or suppression mechanisms of aggressive behavior with negative consequences provided by descending control systems in the prefrontal cortex are both influenced by predicting expectations of reward and punishment as well as by early sensory and social information processing, and early cognitive appraisal [46].

In light of this theoretical model of suicidal process we might hypothesize that a sudden increase of subjectively perceived wish to die might serve more as a provoking trigger stimulus of self-aggression. However, a chronic and sustained self-perceived lack of vigor and vitality might rather interfere with the mechanism of aggressive diathesis as an influencing factor in early processing of other provoking trigger stimuli.

The suggested association of *VI* with an inner balance between subjective experiences of a wish to live versus a wish to die might represent a complementary factor to be assessed and monitored clinically alongside various other components and risk factors for vulnerability to suicidal behavior. The need for better prediction of suicidal risk is particularly important for evaluating persons with a bipolar disorder, with predominantly dysphoric longitudinal morbidity and with impulsive aggressive personality traits, as well as those with psychotic mood disorders which also involve impaired risk-assessment and enhanced proneness to violent behavior.

Bipolar mood disorders are often characterized by particularly severe, unstable, and frequently changing, long-term morbidity leading to functional impairment and increased mortality. Therefore, rational and safe clinical management of depressed patients requires timely differentiation of bipolar and unipolar mood disorders. In particular, it is challenging to differentiate bipolar from unipolar disorders when early episodes present as major depression and a history of mania or hypomania is not available or not recognized, as is particularly likely in bipolar-II disorder patients. We suggest that biological measures such as *VI* might help to limit misdiagnosis and delayed or inappropriate treatments.

Limitations of this study include sample size that was small but sufficient to demonstrate a relationship of *VI* to suicidal ideation and to diagnosis (Table 2). Although it is acknowledged that a small sample size increases the risk of type II error and a power analysis was not conducted prior to this proof of concept study, a post-hoc analysis found statistical power to be adequate. In addition, actigraphy monitoring needs to be repeated through various morbid states and in subjects with versus without previous suicide attempts. However, our previous research has shown that *VI* has both stable (trait-like) and varying (state-like) features [27]. It is possible that the association with suicidal thinking found here has a trait component, and indeed there is external evidence that suicidal behavior has this trait/state nature [47]. .

Additional studies are warranted to assess the stability of the observed association of *VI* levels and suicidal ideation and to extend it to suicidal behaviors and related measures (including intent, plans and actions) through changing clinical states including euthymia as well as in chronically suicide-contemplating clients for suicide risk may lie well beyond observable mood psychopathology. Long-term actigraphic monitoring of psychomotor disturbances might be also included in psychopharmacological studies of clinical depression as an objective correlate of treatment response, as well as a measure to assist the clinical appraisal of emerging suicidal risk when psychomotor arousal precedes mood improvement [45].

In conclusion, the present findings indicate that *VI*, an objective measure derived from analysis of motility rhythms recorded by noninvasive, electronic actigraphy in depressed human subjects, correlated inversely with suicidal ideation or a wish to die, and elevated *VI* scores (≥3.0) distinguished unipolar from bipolar depression with favorable Bayesian characteristics. An objective biomarker of suicidal ideation could be particularly advantageous when patients are unwilling or unable to share suicidal thoughts with clinicians. A patient’s determined suicidal intent, difficulties in self-expression or an unsatisfactory therapeutic relationship are circumstances that would benefit from an objective estimate of suicidal thinking.

## References

[1] Robins E, Guze SB (1970). Establishment of diagnostic validity in psychiatric illness - its application to schizophrenia.. American Journal of Psychiatry.

[2] Schmidt HD, Shelton RC, Duman RS (2011). Functional biomarkers of depression: diagnosis, treatment, and pathophysiology.. Neuropsychopharmacology.

[3] Judd LL, Akiskal HS, Zeller PJ, Paulus M, Leon AC (2000). Psychosocial disability during the long-term course of unipolar major depressive disorder.. Archives of General Psychiatry.

[4] Charlson FJ, Stapelberg NJC, Baxter AJ, Whiteford HA (2011). Should global burden of disease estimates include depression as a risk factor for coronary heart disease?. BMC Medicine.

[5] Baldessarini RJ, Vieta E, Calabrese JR, Tohen M, Bowden CL (2010). Bipolar depression: overview and commentary.. Harvard Review Psychiatry.

[6] Parker G, Fletcher K, Hyett M, Hadzi-Pavlovic D, Barrett M (2009). Measuring melancholia: the utility of a prototypic symptom approach.. Psychological Medicine.

[7] Salvatore P, Baldessarini RJ, Tohen M, Khalsa HMK, Sanchez-Toledo JP (2009). McLean-Harvard international first-episode project: two-year stability of ICD-10 diagnoses in 500 first-episode psychotic disorder patients.. Journal of Clinical Psychiatry.

[8] Parker G (2000). Classifying depression: Should paradigms lost be regained?. American Journal of Psychiatry.

[9] Weinstock LM, Strong D, Uebelacker LA, Miller IW (2010). DSM-IV depressive symptom expression among individuals with a history of hypomania: A comparison to those with or without a history of mania.. Journal of Psychiatric Research.

[10] Oquendo MA, Galfalvy HC, Currier D, Grunebaum MF, Sher L (2011). Treatment of suicide sttempters with bipolar disorder: a randomized clinical trial comparing lithium and valproate in the prevention of suicidal behavior.. American Journal of Psychiatry.

[11] Mann JJ (2003). Neurobiology of suicidal behaviour.. Nature Reviews Neuroscience.

[12] Mann JJ, Arango VA, Avenevoli S, Brent DA, Champagne FA (2009). Candidate endophenotypes for genetic studies of suicidal behavior.. Biological Psychiatry.

[13] Mann JJ, Currier DM (2010). Stress, genetics and epigenetic effects on the neurobiology of suicidal behavior and depression.. European Psychiatry.

[14] Asberg M, Staff DM, Mann JJ (1997). Neurotransmitters and suicidal behavior - The evidence from cerebrospinal fluid studies..

[15] Anguelova M, Benkelfat C, Turecki G (2003). A systematic review of association studies investigating genes coding for serotonin receptors and the serotonin transporter: I. Affective disorders.. Molecular Psychiatry.

[16] Gottesman, II, Gould TD (2003). The endophenotype concept in psychiatry: etymology and strategic intentions.. American Journal of Psychiatry.

[17] Courtet P, Gottesman, II, Jollant F, Gould TD (2011). The neuroscience of suicidal behaviors: what can we expect from endophenotype strategies?. Translational Psychiatry 1: pii,e7.

[18] Salvatore P, Baldessarini RJ, Centorrino F, Egli S, Albert M (2002). Weygandt's on the mixed states of manic-depressive insanity: a translation and commentary on its significance in the evolution of the concept of bipolar disorder.. Harvard Review Psychiatry.

[19] Weiss BL, Foster FG, Reynolds CF, Kupfer DJ (1974). Psychomotor activity in mania.. Archives of General Psychiatry.

[20] Wolff EA, Putnam FW, Post RM (1985). Motor-activity and affective-illness - the relationship of amplitude and temporal distribution to changes in affective state.. Archives of General Psychiatry.

[21] Teicher MH (1995). Actigraphy and motion analysis: new tools for psychiatry.. Harvard Review Psychiatry.

[22] Souetre E, Salvati E, Belugou JL, Pringuey D, Candito M (1989). Circadian-rhythms in depression and recovery - evidence for blunted amplitude as the main chronobiological abnormality.. Psychiatry Research.

[23] Wehr TA, Muscettola G, Goodwin FK (1980). Urinary 3-methoxy-4-hydroxyphenylglycol circadian-rhythm - early timing (phase-advance) in manic-depressives compared with normal subjects.. Archives of General Psychiatry.

[24] Teicher MH, Lawrence JM, Barber NI, Finklestein SP, Lieberman HR (1988). Increased activity and phase delay in circadian motility rhythms in geriatric depression - preliminary-observations.. Archives of General Psychiatry.

[25] Murray G, Allen NB, Trinder J (2003). Seasonality and circadian phase delay: prospective evidence that winter lowering of mood is associated with a shift towards Eveningness.. Journal of Affective Disorders.

[26] Salvatore P, Ghidini S, Zita G, De Panfilis C, Lambertino S (2008). Circadian activity rhythm abnormalities in ill and recovered bipolar I disorder patients.. Bipolar Disorders.

[27] Indic P, Salvatore P, Maggini C, Ghidini S, Ferraro G (2011). Scaling behavior of human locomotor activity amplitude: association with bipolar disorder.. Plos One.

[28] Ivanov PC, Rosenblum MG, Peng C-K, Mietus J, Havlin S (1996). Scaling behavior of heartbeat intervals obtained by wavelet-based time-series analysis.. Nature.

[29] Monk TH (1989). A visual analog scale technique to measure global vigor and affect.. Psychiatry Research.

[30] Gross G, Huber G, Klosterkoetter J, Linz M (1992). Bonner skala fuer die beurteilung von basissymptomen.. Berlin: Springer-Verlag.

[31] Supprian U (1975). Eppendorf mood and drive scale.. Pharmakopsychiatrie Neuro-Psychopharmakologie.

[32] Novick DM, Swartz HA, Frank E (2010). Suicide attempts in bipolar I and bipolar II disorder: a review and meta-analysis of the evidence.. Bipolar Disorders.

[33] Brown GK, Steer RA, Henriques GR, Beck AT (2005). The internal struggle between the wish to die and the wish to live: a risk factor for suicide.. American Journal of Psychiatry.

[34] Scheler M (1973). Formalism in ethics and non-formal ethics of values.. Evanston, IL: Northwestern University Press.

[35] Maggini C, Cassano GB, editors (1982). La depressione. Aspetti sintomatologici e diagnostici attuali.. Milan: Masson e Cie.

[36] Benazzi F (2008). A relationship between bipolar II disorder and borderline personality disorder?. Progress in Neuro-Psychopharmacology and Biological Psychiatry.

[37] Beigel A, Murphy DL (1971). Unipolar and bipolar affective illness - differenced in clinical charecteristics accompanying depression.. Archives of General Psychiatry 24: 215-&.

[38] Kuhs H, Reschke D (1992). Psychomotor activity in unipolar and bipolar depressive patients.. Psychopathology.

[39] Bertocci MA, Bebko GM, Mullin BC, Langenecker SA, Ladouceur CD (2011). Abnormal anterior cingulate cortical activity during emotional n-back task performance distinguishes bipolar from unipolar depressed females.. Psychol Med.

[40] Versace A, Almeida JRC, Quevedo K, Thompson WK, Terwilliger RA (2010). Right orbitofrontal corticolimbic and left corticocortical white matter connectivity differentiate bipolar and unipolar depression.. Biological Psychiatry.

[41] Almeida JRC, Versace A, Hassel S, Kupfer DJ, Phillips ML (2010). Elevated amygdala activity to sad facial expressions: a state marker of bipolar but not unipolar depression.. Biological Psychiatry.

[42] Amsterdam JD, Newberg AB (2007). A preliminary study of dopamine transporter binding in bipolar and unipolar depressed patients and healthy controls.. Neuropsychobiology.

[43] Cannon DM, Ichise M, Rollis D, Klaver JM, Gandhi SK (2007). Elevated serotonin transporter binding in major depressive disorder assessed using positron emission tomography and [C-11]DASB; Comparison with bipolar disorder.. Biological Psychiatry.

[44] Rao U, Dahl RE, Ryan ND, Birmaher B, Williamson DE (2002). Heterogeneity in EEG sleep findings in adolescent depression: unipolar versus bipolar clinical course.. Journal of Affective Disorders.

[45] Ayd FJ (1961). Recognizing the depressed patient: with essentials of management and treatment.. New York: Grune & Stratton.

[46] Siever LJ (2008). Neurobiology of aggression and violence.. American Journal of Psychiatry.

[47] Lee BH, Kim YK (2011). Potential peripheral biological predictors of suicidal behavior in major depressive disorder.. Progress in Neuro-Psychopharmacology and Biological Psychaitry.

